# EMT Regulation by Autophagy: A New Perspective in Glioblastoma Biology

**DOI:** 10.3390/cancers11030312

**Published:** 2019-03-06

**Authors:** Barbara Colella, Fiorella Faienza, Sabrina Di Bartolomeo

**Affiliations:** 1 Department of Biosciences and Territory, University of Molise, 86090 Pesche (IS), Italy; b.colella@studenti.unimol.it; 2 Department of Biology, University of Rome Tor Vergata, 00133 Rome, Italy; fiorella.faienza@uniroma2.it

**Keywords:** autophagy, epithelial-to-mesenchymal transition (EMT), glioblastoma (GBM), cadherins, Wnt/β-catenin signalling

## Abstract

Epithelial-to-mesenchymal transition (EMT) and its reverse process MET naturally occur during development and in tissue repair in vertebrates. EMT is also recognized as the crucial event by which cancer cells acquire an invasive phenotype through the activation of specific transcription factors and signalling pathways. Even though glial cells have a mesenchymal phenotype, an EMT-like process tends to exacerbate it during gliomagenesis and progression to more aggressive stages of the disease. Autophagy is an evolutionary conserved degradative process that cells use in order to maintain a proper homeostasis, and defects in autophagy have been associated to several pathologies including cancer. Besides modulating cell resistance or sensitivity to therapy, autophagy also affects the migration and invasion capabilities of tumor cells. Despite this evidence, few papers are present in literature about the involvement of autophagy in EMT-like processes in glioblastoma (GBM) so far. This review summarizes the current understanding of the interplay between autophagy and EMT in cancer, with special regard to GBM model. As the invasive behaviour is a hallmark of GBM aggressiveness, defining a new link between autophagy and EMT can open a novel scenario for targeting these processes in future therapeutical approaches.

## 1. Introduction

Glioblastoma (GBM) is the most malignant and frequent form of glioma. This brain tumor is derived from glial cells and is characterized by high proliferation rate and local dissemination. Despite the improvement of chemo- and radio-therapies obtained in last decades, GBM prognosis is very poor with a median survival time that rarely exceed 18 months. Notably, GBM is characterized by resistance to apoptosis and high invasiveness, driving the search for novel targets useful to design effective therapeutical strategies.

The Epithelial-to-Mesenchymal Transition (EMT) programme is considered to be crucial for the acquisition of an invasive phenotype in epithelial cancer cells. Although some differences have been outlined, glioma cells also undergo an EMT-like process, through the activation of specific transcription factors and signalling pathways. Considering the critical role played by EMT on GBM dissemination, resistance to apoptosis and maintenance of cancer stem cells staminality, a number of preclinical studies have been launched to target the process as therapeutic approach.

In addition, the role of autophagy in tumor onset, progression is acquiring increasing clinical inerest. For instance, it has been recently shown that, besides modulating cell resistance or sensitivity to therapy, autophagy can also modulate GBM invasion.

In this review, we examined the current understanding of the role of autophagy in regulating the EMT and EMT-like programmes and in directing GBM cells to a more or less invasive phenotype. Moreover, we provided some clues to argue that blocking autophagy for therapeutic purposes requires careful consideration.

## 2. Epithelial-to-Mesenchymal Transition

EMT is a biological process by which epithelial polarized cells undergo various biochemical modifications that convert them in mesenchymal, isolated, and not-polarized cells. A lot of evidence suggests that EMT and its reverse process (mesenchymal-to-epithelial transition, MET) are crucial for tissue remodeling during development, wound repair and the initiation of cancer metastasis. In the early 1980s, Elizabeth Hay described epithelial-to-mesenchymal phenotype changes in the primitive streak of chick embryos [1]. Initially named as “epithelial to mesenchymal transformation”, this process is now known as “epithelial-to-mesenchymal transition” to emphasize its transient nature and to distinguish it from the neoplastic transformation. To acquire a mesenchymal phenotype, epithelial cells undergo morphological and biochemical changes, reorganize their cytoskeleton, and activate a specific transcriptional programme. Indeed, epithelial cells are characterized by an apical-basal polarity, a polygonal shape and various kind of junctions. The latter tightly holds epithelial cells against each other and anchors them to the basement membrane, thus ensuring the structural integrity of epithelial sheets within the body tissues [2]. Conversely, mesenchymal cells exhibit a spindle-like morphology, do not have any polarity or connection with other and they are loosely anchored to the extracellular matrix (ECM) by focal adhesions.

Transcription factors (TFs) belonging to the SNAI family, named Snail and Slug, the zinc-finger E-box-binding homeobox (ZEB)1/2, and Twist1/2 are recognized to be the master regulators of EMT execution, since they promote the transcription of genes normally expressed in mesenchymal cells, such as N-cadherin, vimentin, and fibronectin. On the contrary, they suppress the expression of the epithelial markers E-cadherin, claudins, occludins, and cytokeratins. Loss of E-cadherin, in turn, promotes Wnt signaling and β-catenin accumulation in the nucleus, where it activates Tcf/LEF-dependent transcription of genes promoting proliferation and migration [3].

Cells undergoing EMT lose their apical–basolateral polarization and acquire a fibroblast-like morphology that allow them to degrade the underlying basement membrane and to migrate from the epithelial layer in which they originated [4]. Metalloproteases are also activated during EMT, and favor cell migration by degrading the membrane basement and the extracellular matrix components [2].

Furthermore, it has been shown that noncoding microRNAs play a role in EMT, by regulating the translation of EMT players, as extensively addressed in Abba et al. [5].

EMT can be classified in three different subtypes according to EMT meetings discussion: the so-called “type 1” EMT occurs during implantation, embryogenesis and organogenesis, the “type 2” EMT is associated to wound healing, tissue regeneration and organ fibrosis, and the “type 3” EMT characterizes neoplastic cells during metastatization. For a comprehensive overview of type 1 and type 2, refer to Kalluri et al., 2009 [4]. The activation of a “type 3” EMT programme (hereafter referred to as EMT) has been proposed to be pivotal for the acquisition of a malignant phenotype by cancer cells [6], as discussed below in detail.

### 2.1. Signals Stimulating Epithelial-to-Mesenchymal Transition in Cancer

Epithelial carcinoma cells, and typically those present at the external part of the tumoral mass, can acquire a mesenchymal phenotype upon specific stimuli. The microenvironment surrounding the primary tumor (TME) is characterized by inflammation, hypoxia, ECM components and tissue-specific soluble factors [7]. Notably, tumor cells recruit activated fibroblasts and immune cells that secrete cytokines, that, in turn, can activate the EMT programme. TGF-β is secreted by stromal fibroblasts, platelets and tumor cells themselves, and is considered the main EMT activator as it induces the expression of specific TFs in different cancer models [8,9,10].

Among cytokines, Tumor Necrosis Factor-α (TNFα) is crucial for EMT induction, and its effects are mediated through NFκB signaling pathway activation [11,12,13]. It has been also described that interleukins, particularly IL6, released by TME cells, can contribute to EMT stimulation. Other EMT-inducing signals originated from the tumor stroma are represented by growth factors such as HGF, EGF, PDGF that are able to activate EMT-specific TFs [14,15,16,17].

Furthermore, tumor microenvironment is characterized by hypoxia that promotes EMT via hypoxia-inducible-factor-1α (HIF1α) activation [18,19]. HIF1α stimulates inflammatory cytokines [20] and cooperates with Wnt/β-catenin signaling to enforce the EMT induction [21]. Moreover, during hypoxia, mitochondria increase the production of reactive oxygen species (ROS) that further contribute to EMT activation via both stimulating NFκB signaling [22], ECM regulation and cytoskeleton remodeling [23].

Once they have acquired a mesenchymal phenotype, cancer cells can dissociate from the primary mass, migrate and eventually enter the blood vessels by intravasation to initiate the metastatic process. Following extravasation and micrometastases formation, invading cells activate a MET programme and form macroscopic metastases resembling the epithelial features of the originating primary tumor, although the molecular mechanisms of MET have been less investigated if compared to those regulating EMT. However, both EMT and MET activation seem to be highly tissue-specific and strictly dependent on the local microenvironment encountered [2,6,24,25].

### 2.2. Epithelial-to-Mesenchymal Transition in Glioblastoma Dissemination

EMT has been mainly characterized in carcinoma models, and the role of EMT in glioma has only recently been investigated [26,27,28]. Candidate cells for originating gliomas (cells of origin) are mostly neural stem cells (NSCs), normally present in the adult brain, and oligodendrocyte precursor cells (OPCs) [29,30,31,32]. However, the involvement of more differentiated cells cannot be completely excluded [33]. As a consequence of the neurodevelopmental process, neural cells assume a mesenchymal phenotype, different from the epithelial one typical of the ectodermal cells they derive from. Therefore, gliomas do not undergo the classical EMT programme during tumorigenesis, and the terms “EMT-like” or “glial-to-mesenchymal transition (GMT)” have been proposed to indicate this peculiar process [34]. Although it is not clear whether cells of origin undergo or not significant modifications toward more mesenchymal features, glioma cells show a high plasticity in terms of EMT-like/MET-like conversion, likely mediated by epigenetic alterations induced by the tumor microenvironment [35,36]. Based on The Cancer Genome Atlas (TCGA) classification, GBMs can be differentiated into four genetic subtypes: Mesenchymal, Classical, Neural and Proneural [37]. Verhaak results suggest that Proneural GBM patients do not have a survival advantage from aggressive therapeutical treatments, unlike Classical and Mesenchymal GBM patients [37]. Role of EMT in each GBM genetic subtype has been investigated by Zarkoob et al. in 2013 [38]. A significant overlap between the genes that are up-regulated in the EMT signature and those that are up-regulated in each of the GBM subtypes exists, although, among all, the mesenchymal subtype has the highest number and expression levels of up-regulated genes [38]. Indeed, GBMs belonging to the mesenchymal subtype are characterized by an elevated invasive potential, poor clinical prognosis, and significantly shortened time to recurrence following initial treatment, compared to the other subtypes. The most commonly used glioma cell lines also present a predominant mesenchymal signature.

GBM cells in the invasive front, differently from those of the inner mass, commonly execute an invasion programme characterized by detachment from the mass, direct adhesion and degradation of ECM (lack of basement membrane) and widespread dissemination in the surrounding brain tissue. Remarkably, unlike other tumors, GBMs only rarely form metastases outside CNS, even though a hallmark of their aggressiveness is the infiltration and the diffuse growth in the surrounding parenchyma.

Large-scale genetic analyses have suggested that signaling networks activated during the physiological neural development are also employed by GBM cells to promote tumor growth and invasion [39,40,41]. In detail, pathways mediated by Wnt/β-catenin, TGF-/β, Tyrosine kinase receptors and SDF/CXCR4 have been involved in the activation of EMT-like related genes to promote GBM dissemination [26,27]. Kahlert et al. found that the Wnt/β-catenin pathway is predominantly activated within cells located at the invasive peritumoral front of patient specimens belonging to the mesenchymal subtype. Chiefly, it induces the expression of Zeb1, Twist1 and Slug, thus promoting the migratory capability of GBM cells in vitro [42].

Regarding TGF-β pathway, a number of evidence demonstrated its critical role for the promotion of invasive properties of glioma cells [43,44,45], although the molecular mechanisms involved need to be further investigated. Interestingly, TGF-β signaling is known to be crucial in the maintenance of the mesenchymal stem-like population in GBM [45,46]. ZEB1 seems to be the pivotal mesenchymal transcription factor activated by TGF-β signaling since, differently from Snail, Slug and Twist, it accumulates in the nucleus of GBM cells [47].

The Hepatocyte growth factor (HGF) binding to the tyrosine kinase receptor c-MET is another crucial event highly activated. c-MET is overexpressed within GSC populations [48] and in patient-derived GSCs belonging to the mesenchymal subtype [49]. Accordingly, elevated c-MET signaling enhances GSC migration by activating EMT TFs [49,50] and is associated with poor survival and increased tumor invasiveness in patients [51,52,53].

The majority of molecules involved in the classical EMT have also been shown to play also a role in the EMT-like process. An increased activity of the TFs that mainly orchestrate the typical EMT, such as SNAI proteins, ZEB1/2, and Twist, promotes the invasion of GBM cells [54,55,56,57]. For instance, SNAIL silencing reduces invasion, migration and proliferation in GBM cell lines [58,59] and overexpression of Slug correlates with GBM grade [56]. ZEB1 and ZEB2 expression is also correlated with invasive features and with survival of GBM patients; ZEB1 knock-down cells formed less invasive and more drug-sensitive masses than wild type cells when inoculated in mouse brain [54,60]. Moreover, Twist1 and Twist 2 expression, besides affecting stemness properties, has been associated to the invasive properties of GBM cells as it mediates the expression of crucial EMT-related genes such as metalloproteinase 2 (MMP2), Slug and HGF among others [57].

It is worth mentioning that the classical cadherin switch, which is widely accepted as an EMT hallmark in carcinomas, is a controversial matter in GBM. Differently from carcinomas, E-cadherin expression is almost absent in neural tissues, where its expression appears limited to GCSs cells and to a subset of highly aggressive GBM cells. Otherwise, N-cadherin is absent in epithelial tumors before the EMT execution, whereas is highly expressed in astrocytes, where it contributes to regulate cell polarity and migration and in GBM cells, that show a faster and less-directed movement to respect to astrocytes [54,61]. It was found that N-cadherin expression is inversely correlated with the invasive behaviour of GBM, and its ectopical expression reduces cell migration and restores polarity in GBM cells [62,63]. Notably, it has also been shown that differences in N-cadherin distribution rather than in its expression levels are responsible for different motility behaviours [64,65].

In addition, several studies showed that the treatment of primary GBMs with radiation therapy or with the anti-angiogenic agents Bevacizumab promotes the acquisition of a mesenchymal phenotype in recurrent tumors [34,66,67,68]. Indeed, glioma cells that have acquired radioresistant properties following treatment exhibit a gene expression profile enriched for genes involved in EMT-related processes [34,69,70], and the pathways promoting EMT result strongly upregulated in these cells, thereby resulting in an increased invasion capability [71,72]. An in vivo study by Halliday et al. demonstrated that proneural GBM cells rapidly shifted their gene expression pattern towards a mesenchymal phenotype in response to radiation therapy in a tumor-bearing mouse model [66]. As radiation is a universal component in the treatment of GBMs, this subtype shift poses an important clinical challenge, especially considering that cells shifted to a mesenchymal subtype display an increased radioresistance [73]. If this shift is due to changes in the microenvironment or to clonal selection of mutant therapy-resistant cells is controversial, but both the hypotheses seem to be possible.

## 3. Autophagy

The term “autophagy” was coined by the discoverer of lysosomes Christian de Duve and it means, in Greek language, “self-eating” [74,75,76]. From the first description of the process in 1960s, many studies described the process of self-degradation by a morphological point of view, until in 1993 a genetic screen led to isolation of some yeast autophagy-defective mutants and to identification of the so-called AuTophaGy-related (ATG) genes [77]. This seminal work allowed Yoshinori Oshumi to be awarded the Nobel Prize in Physiology and Medicine in 2016. Oshumi’s screen identified 15 genes involved in autophagy regulation in yeast undergoing nutrient deprivation, but today, more than 40 genes have been described in yeast, many of them having orthologues in vertebrates [76]. Three main types of autophagy have been described: macroautophagy, microautophagy and Chaperone-Mediated Autophagy (CMA).

### 3.1. Mechanisms and Molecules

During macroautophagy (hereafter referred to as autophagy), double-membrane vesicles named autophagosomes form and engulf cytoplasmic material, including long-lived proteins and old or damaged organelles which are then delivered to lysosomes for degradation and recycling [78]. Always activated at basal level within the cell, autophagy can be modulated by several signals, mainly by nutrient signaling, growth factors, energy status, oxidative or ER stress and pathogen infection. The input from these upstream signals is integrated by the serine/threonine protein kinase mTOR (mechanistic or mammalian target of Rapamycin), which acts upstream of the ATG genes, thus controlling autophagy activation [79]. mTOR belongs to the phosphoinositide 3-kinase (PI3K)-related kinase family, and, in mammalian cells, works as the catalytic subunit of two multi-protein complexes known as mTORC1 and mTORC2 [80]. Under nutrient deprivation, mTOR is inhibited and Ulk1/Atg13-FIP200 complex initates and drives a massive autophagy activation [81]. Autophagosome formation requires the formation of a multi-protein complex, composed by class III PI3K, Beclin1 and p150, although other proteins such as UVRAG, Ambra1 and Atg14L are able to bind and regulate the complex [82,83,84,85,86]. Elongation and maturation of autophagosomes involve two ubiquitin-like conjugation systems, both requiring Atg7, which catalyze the covalent linkage of ATG5 to ATG12 and ATG16-like 1, and the attachment of phosphatidylethanolamine to proteins of the microtubule-associated protein 1 light chain 3 (MAP1LC3 or LC3) family [87].

Lipidated LC3 protein is then recruited to the autophagosome membrane that docks and fuse with lysosome, resulting in the formation of a single membrane vesicle named autophagolysosome or autolysosome. Lysosomal hydrolases, ultimately, degrade and recycle the content of autolysosomes.

Although originally suggested to be just a nonspecific and bulk degradation mechanism, autophagy is now recognized as a highly regulated process, enabling cells to sense and promptly respond to a plethora of stimuli, thereby conferring adaptation to the ever-changing environment. Nevertheless, even though a basal level of autophagy contributes to maintain the proper cell homeostasis both during embryogenesis and adulthood in physiological conditions, it is now ascertained that autophagy is deregulated in various human pathologies, including cancer [88,89,90].

### 3.2. Autophagy Role in Tumorigenesis

The observation in 1999 that the gene encoding Beclin1 is monoallelically deleted in a high percentage of human breast, ovarian and prostate cancers provided the first evidence of the involvement of the autophagic process in tumorigenesis [91]. Disruption of Beclin1 in mice results in an increased proliferation and in the spontaneous development of various malignancies, confirming Beclin1 as an haploinsufficient tumor suppressor gene [92,93]. In a similar way, mice lacking one copy of the Beclin1 interactor Ambra1 exhibit a higher incidence of spontaneous tumors than their wild type littermates, and cells depleted of the gene are characterized by a hyperproliferative phenotype [94]. Notably, *Ambra1* homozygous disruption in mouse leads to a strong hyperproliferation and lethal defects in the developing nervous system during embryogenesis [82]. Mice bearing systemic or tissue-specific deletion of Atg5 and Atg7 also develop tumoral masses a higher frequency than the wild type counterparts [95] and are more prone to develop cancers upon carcinogen-induced stimuli [96,97,98].

Several mechanisms have been proposed to explain the oncosuppressive functions of autophagy [90]. First of all, the autophagy-mediated clearance of proteins and organelles ensures the proper cellular homeostasis, avoiding the accumulation of genotoxic molecules, such as reactive oxygen species (ROS) produced by dysfunctional mitochondria, as well as aggregates of ubiquitinated proteins [99,100]. An intact autophagic machinery is also required to deal with cytotoxic stress and to maintain genome stabilization, although further investigation is required to underlie the mechanisms involved [101,102]. Moreover, autophagy counteracts the metabolic switch accompanying malignant transformation by eliminating old and damaged mitochondria, thus preserving the optimal bioenergetic needs and maintaining the physiological metabolic homeostasis [103,104].

Other potential mechanisms through which autophagy acts as an oncosuppressive process are linked to its role in the regulation of immune response [105], maintenance of the staminal niches [106], defens of the organism against pathogen infections and degradation of oncogenic proteins, like mutant (but not wild-type) TP53 [107].

On the other hand, it is well accepted that, in an established tumor, cancer cells use autophagy as a strategy to overcome microenvironmental stresses, including nutrient deprivation, hypoxia and drugs. Advanced tumors sometimes exhibit an increased autophagic flux and ex-vivo cell lines in which BECN1 or ATG5 have been down-regulated are virtually unable to survive within the metastatic niche [108]. Analogously, autophagy-defective tumoral cells appear more sensitive to pro-apoptotic stimuli than autophagy-proficient cells [109,110,111,112].

Due to this dual function, autophagy has been defined a ‘Janus-faced’ player in cancer progression [113]: in the early stages of tumorigenesis it plays onco-suppressive functions by limiting cell proliferation, DNA damage and tumor progression; on the contrary, when the tumor mass is established, it helps cells to counteract the stressful conditions characterizing the tumor microenvironment.

### 3.3. Autophagy and Glioblastoma: Friends or Foes?

It was demonstrated that high-grade gliomas exhibit lower expression of some autophagy related proteins with respect to low-grade ones, and that the progression of astrocytomas toward higher grades is accompanied by a decrease in autophagic proficiency. Pirtoli et al. observed that both BECN1 mRNA (encoding for Beclin1) and protein levels are lower in GBM tissue than in low-grade and healthy brain tissue [114]. Accordingly, following Karnofski classification, high Beclin1 levels have been positively correlated with patient survival and performance status, whereas low Beclin1 expression correlates with an increase of proliferation [114]. Similarly to Beclin1 expression, also LC3B II expression (index of autophagy activation) is low in high-grade astrocytomas, thus suggesting an impairment of the autophagic process in these tumors [115]. On the other hand, in 2012, through a proteomic screening, Galavotti et al. found that some genes involved in autophagy regulation are highly expressed in the GBM mesenchymal subtype [116]. Among these, the autophagy associated genes DRAM1 and SQSTM1 encoding for the key regulator p62 are highly expressed in Glioma stem cells (GSCs), and modulate their migration and invasion capabilities [116]. Although these studies suggest that autophagy may regulate gliomagenesis, a systematic and comprehensive investigation of autophagy role among the GBM subtypes is missing, but needed. Indeed, a different expression of autophagy regulators across GBM genetic groups could be responsible for a different susceptibility to autophagy modulation.

In addition to the growing evidences showing a direct involvement of autophagy-regulating genes in GBM progression, several autophagy-associated molecules are frequently altered in brain tumors. As an example, the tyrosine kinase EGF receptor is often amplified in gliomas, and suppresses autophagy through both kinase-dependent and -independent mechanisms [117]. PTEN, is commonly mutated in gliomas, and positively regulates autophagy by inhibiting the PI3K/Akt pathway, although PTEN and NF1 co-mutation determines an autophagy suppression through the hyperactivation of the PI3K/Akt signalling [118].Furthermore, the oncosuppressor p53, frequently mutated in gliomas, may have a dual role in autophagy regulation, as the nuclear protein is able to promote autophagy through the transcriptionally regulation of autophagy-related genes, whereas cytoplasmic p53 suppresses autophagy [119]. Further investigation are needed to define whether autophagy machinery may be considered as a novel useful prognostic and/or therapeutic marker of glial tumors.

## 4. Autophagy and Epithelial-to-Mesenchymal Transition

**Figure 1:**
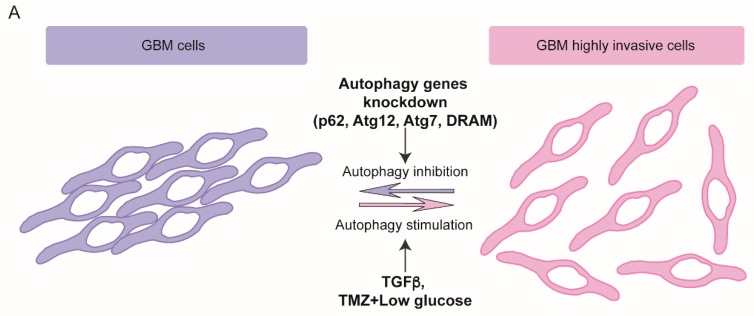

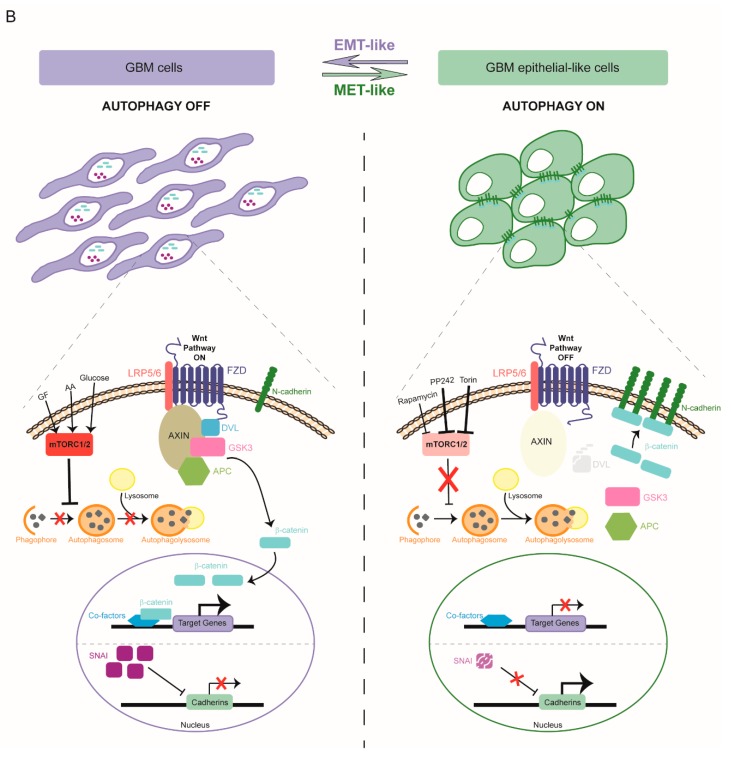
Effects of autophagy modulation on GBM migration/invasion capabilities from opposite point of views. (**A**) Autophagy induction promotes GBM cells invasiveness and viceversa. Upon different stimuli, autophagy is activated and a more invasive phenotype is observed in some models of GBM cells (right). Conversely, when autophagy is impaired by genetic knockdown of some autophagy-related genes, a less invasive phenotype is obtained (left). TGFβ, Tumor necrosis factor β; TMZ, Temozolomide. (**B**) Autophagy modulation promotes EMT/MET-like shifts in GBM cells. In nutrient-rich conditions, hyperactivation of the mTORC1/2 complexes impairs autophagy and Wnt pathway is active thus allowing β-catenin translocation to the nucleus where it promotes the transcription of pro-invasive molecules. In this condition, EMT players of the SNAI family express and repress cadherins expression. The genetic knockdown of autophagy related genes exacerbates the mesenchymal phenotype and enhances the cell migration capability. Upon autophagy induction, shown on the right, Dishvelled (DVL) is degraded and Wnt pathway inactivated leading to β-catenin accumulation into the cytosol. In autophagic cells, SNAI factors are down-regulated and, consequently, N-cadherin accumulates and binds β-catenin, thus promoting cell-cell adhesion.

Autophagy was only recently connected to EMT. In the last years, some observations indicated that an intricate relationship exists between these two important processes in cancer [120]. According to its dual role in tumorigenesis, the effect of autophagy on EMT appears controversial and strictly dependent on the cellular type and on the stimulus employed for activating or inhibiting autophagy, as summarized in Table 1 and in Figure 1.

Literature data highlights that an autophagy stimulation of metastatization could be merely the consequence of its pro-survival activity against the apoptotic signals coming from changes in adhesion and cytoskeleton reorganization [108]. A number of compounds or microenvironmental conditions that are able to activate the EMT programme, can also induce an autophagic response in different types of cultured cancer and non-cancerous cells; autophagy inhibition in these models impairs EMT (Table 1).

However, emerging evidence also indicates that autophagy activation can induce a reversion of the EMT phenotype in different healthy and cancer models and that several anticancer compounds that induce autophagy also inhibit EMT [121,122,123] (Table 1). Selective degradation of EMT players seems to be a general mechanism by which autophagy can modulate EMT process [124]. Notably, in ATG3, ATG5, ATG9 and ATG12 KO mice, p62 accumulation accumulation determines stabilization of Twist1, which is normally degraded by both proteasome and autophagosomes [125]. This regulation can be crucial in those tumors characterized by p62 up-regulation, as observed so far in human squamous cell carcinoma and in melanoma (Table 1). Autophagy deficiency reduces the expression of epithelial markers and increases that of mesenchymal ones also in ATG7 KO hepatocytes [126] (Table 1).

Taken together, these observations show a complex crosstalk between autophagy and EMT processes. It is conceivable that, at the early stages of metastatization, autophagy acts as oncosuppressive signal, tending to inhibit the EMT programme mainly by destabilizing EMT crucial players. Later on, metastatic cells could require a sustained autophagy to survive to environmental and metabolic stressful conditions encountered [113].

### 4.1. Autophagy Role on Glioblastoma Dissemination

To date, only a few studies correlate autophagy to GBM cells capability to migrate and spread toward surrounding tissues, and, similarly to what observed in other tumor models, they highlight two opposite point of views, as detailed below, in Section 4.1.1, and as illustrated in Figure 1.

#### 4.1.1. Autophagy Promotes Glioblastoma Dissemination

In 2012, Galavotti et al. showed that some autophagy players are up-regulated in GBM mesenchymal subtype, and that their modulation modifies the migration properties of GBM cells. They observed that GSCs require autophagy activation to migrate, and down-regulation of the autophagy-associated factors DRAM1, p62 and ATG7 limit their invasion capabilities potentially through the regulation of energy metabolism [116] (Figure 1A). In line with these observations, a study conducted by using an 3D organotypic model of GBM showed that ATG12 RNA silencing reduced cellular invasion, although no modifications of cellular migration capabilities was observed [142] (Figure 1A). More recently, another couple of studies have correlated autophagy activation induced by a combination of different stimuli with an enhanced mesenchymal phenotype in GBM cells through various mechanisms [143,144]. Lastly, inhibiting the late stages of autophagy by using Chloroquine, Liu et al. showed a potentiation of the effect of the multi kinase inhibitor Sorafenib in reducing cell proliferation and migration, through a further stimulation of apoptosis [145] (Figure 1A).

#### 4.1.2. Autophagy Impairs Glioblastoma Dissemination

In spite of the previous mentioned evidence, we and other groups have recently demonstrated a direct effect of autophagy modulation on migration and invasion capabilities of GBM cells, as illustrated in Figure 1B. Autophagy induction by nutrient deprivation or by mTOR inhibition determines a reversion of EMT phenotype in immortalized and primary GBM cells [146,147,148,149]. In 2014, Palumbo et al. observed that ATG7 RNA silencing restored clonogenic ability of irradiated GBM cells with inactive EGFR and, conversely, that rapamycin-mediated autophagy further impaired their clonogenic and migration capabilities [148]. Later on, we demonstrated that autophagy induction in GBM immortalized and primary cells, obtained by nutrient starvation or by mTOR pharmacological inhibition, induced a drastic impairment of both migration and invasiveness. On the contrary, autophagy deficiency, obtained by silencing the autophagy master genes ATG5, ATG7 or BECN1, stimulated cell motility [146], similarly to what observed in highly metastatic breast, colon and hepatocellular cancer models [122,124,130]. We correlated the migration properties of the cells analyzed with a molecular shift from a mesenchymal to an epithelial-like phenotype (Figure 1B). Upon autophagy induction, in fact, we found a down-regulation of the EMT players Snail and Slug, likely due to a general impairment of protein synthesis mediated by mTOR inhibition, rather than to the autophagosome-mediated degradation [146]. As an outcome of SNAI down-regulation, the up-regulation of N- and R-cadherin mRNA and protein expression was observed, whereas no significant differences in other cadherin family members were found (Figure 1B). Remarkably, as above discussed, although a “cadherin shift” from the E- to the N- isoform is actually believed a hallmark of carcinoma cells undergoing EMT, the role of cadherins in non-epithelial tumors is much less documented and elucidated. N-cadherin overexpression or re-distribution has been associated with the recovery of the cell polarity and the inhibition of migration of GBM cells [62,63,64,65]. Notably, we have recently found that, upon autophagy induction, the Wnt signaling effector β-catenin relocalises within the cell and associates to N-cadherin in sub-membrane compartments to form epithelial-like cell-cell adhesion structures [147], thus contributing to a mesenchymal-to-epithelial-like transition of GBM cells (Figure 1B). Similarly to other tumors, Wnt pathway is constitutively active in GBM and its deregulation is likely responsible for initiation and/or progression of the disease [150,151,152,153]. β-catenin translocation to the nucleus characterizes the mesenchymal and invasive phenotype of tumoral cells as it promotes SNAIL and ZEB1 transcription and mediates EGFR pathway-induced EMT [154,155,156].

In summary, a complicated interplay between autophagy process and EMT/MET activation in GBM is recently emerging. Contradictory results could be explained by the different stimuli and different models employed. Notably, autophagy induction obtained through mTOR inhibition or nutrient deprivation always results in migration/invasion reduction in independent experiments and different GBM cell lines. Conversely, autophagy activation resulting by the employment of drugs like TMZ plus low glucose, or by TGF-β, or by AEG overexpression is associated to promotion of migration capabilities of GBM cells, maybe suggesting that different and complex mechanisms contribute to the migratory phenotype in those contexes.

Effects observed following autophagy inhibition also require attention, although Chloroquine effects on cells often depend on autophagy-independent mechanisms, as better illustrated in the next section. More controversial, although conducted in different model systems, are the results on GBM invasiveness obtained by inactivating ATG genes. In light of such contradictory results, further in-depth investigation is, for sure, required to better unravel the question.

### 4.2. Targeting Autophagy in Glioblastoma Therapy

**Figure 2:**
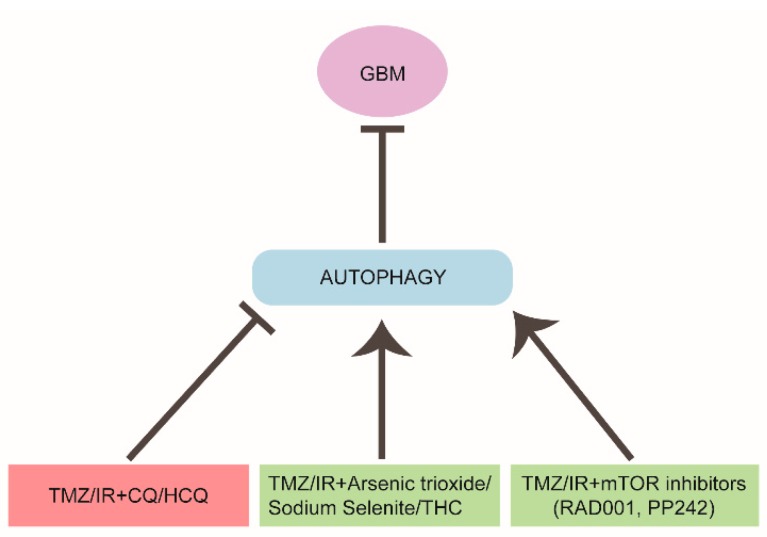
Targeting autophagy in GBM therapy. The effects of different therapeutic combinations on autophagy and the outcome on GBM progression in patients are shown. Chloroquine or hydroxy-chloroquine (CQ/HCQ) addition to standard protocols (TMZ/IR, temozolomide/radiation therapy) impairs the autophagy flux and sensitizes GBM to the treatment (pink box). Otherwise, several chemical compounds and mTOR inhibitors, that are able to stimulate autophagy, also increase the efficacy of the standard treatments (green boxes). THC, tetrahydrocannabinol.

In the last decade, the standard therapeutic regimen for GBM has been surgical resection, if feasible, followed by radiation therapy (IR) and temozolomide (TMZ)-based chemotherapy. Both TMZ and IR are able to induce an autophagic response in GBM cell lines, likely through DNA damage induction, but the outcome of the autophagic response is so far inconclusive (Figure 2). In 2004, Kanzawa et al. reported that TMZ treatment induced autophagy instead of apoptosis in GBM cells and that the co-treatment with a pharmacological inhibitor of the late stages of autophagy restored TMZ-induced cytotoxicity, although the opposite effect was obtained by blocking autophagy initiation [157]. In line with this observation, the combination between standard therapies and autophagy flux inhibitors Chloroquine (CQ) or its analog hydroxychloroquine (HCQ) is a promising therapeutic approach in GBM treatment, and combined therapies including these molecules are currently employed in ongoing phase III clinical trials (Figure 2). Indeed, CQ and its derivatives are, in fact, the only autophagy inhibitors already approved by the USA Food and Drug Administration due to their anti-inflammatory and anti-malarial properties. Similar to other tumoral settings, CQ-treated GBM patients exhibit a better median survival if compared to placebo-treated individuals [158]. However, despite the potential promising beneficial effect, a trial combining HCQ with TMZ and IR showed that the maximum tolerated HCQ dose was ineffective in inhibiting autophagy, suggesting that CQ effect could be due to autophagy-independent mechanisms [159]. Notably, it has been recently shown that, in addition to DNA intercalation properties and ROS production, CQ also induces a strong autophagy-independent disorganization of the Golgi apparatus and of the endo-lysosomal system in mice tissues, thus suggesting caution in interpreting results obtained with this drug [160,161].

In an apparently contradictory way, other therapeutic approaches and clinical trials aimed at inhibiting mTOR pathway, inducing in turn an autophagic response, have been launched. PI3K/Akt/mTOR pathway is often deregulated in human cancers including GBM, and is involved in cancer stem cell maintenance, thus inducing an uncontrolled proliferation [162,163]. It was observed that the co-treatment of TMZ and the mTOR inhibitor RAD001 (also known as everolimus) induced cell death [164] (Figure 2). RAD001 was shown to enhance the cytotoxic effect of an oncolytic adenovirus in a viral-mediated therapy by inducing an autophagy-dependent cell death [165,166].

mTOR inhibition also promotes TMZ-induced senescence in in vitro and in vivo models [167,168]. However, the link between autophagy and senescence needs to be further clarified in order to be exploited a potential therapeutic tool.

Very recently, it has been suggested that the failure of some clinical trials targeting PI3K and mTOR, could be due to the employment of Rapalogs (rapamycin and its analogs) which are known to inhibit mTORC1, but not mTORC2 [149]. In fact, a feedback mechanism activated by mTORC1 inhibition stimulates mitogenic pathways, thus compromising the rapalog efficacy on cell proliferation [169]. In order to overcome the emerged limitations, a second generation of mTOR inhibitors, (named ATP-competitive mTOR kinase inhibitors or TORKIs) have been developed and are revealing more efficacious than rapalogs in GBM treatment [170,171,172]. As an example, the novel TORKI PP242, able to target both mTORC1 and mTORC2, impairs cell proliferation and reduces stemness and invasiveness properties in a group of GBM cell lines carrying different genetic alterations [149] (Figure 2). In this respect, a putative difference in the “autophagy signature” among GBM subtypes, if found, could affect the response to the treatments.

Notably, in our model, we have obtained β-catenin relocalisation and migration impairment by both nutrient deprivation and by inhibiting mTOR complexes by means of Torin 1, an ATP-competitive mTOR inhibitor able to target both the mTOR complexes [147].

Finally, a number of compounds, such as arsenic trioxide, sodium selenite and cannabinoids (THC), used in combination with traditional therapy showed beneficial effects through the induction of an autophagic response, in some cases potentiating drug-induced cell death, some other inducing mitochondrial damage or ER stress [173,174,175,176] (Figure 2). In addition, autophagy-induced cell death seems also to be the mechanism by which some compounds overcome the apoptosis-resistance typical of the anoxic cells inside tumors. For instance, the class of small molecules able to bind the BH3 domain of the anti-apoptotic protein Bcl-2, known as BH3 mimetics, were found to induce an autophagy-dependent cell death in GBM [177,178]. If these compounds are also able to trigger a beneficial or detrimental effect on GBM invasiveness remains to be investigated.

## 5. Concluding Remarks

GBM is the most common and aggressive brain malignancy, and is characterized by a highly invasive behaviour, although the role of EMT in GBM dissemination has only been recently investigated. Although several molecules and signalling pathways mediating EMT in carcinomas play a role also in glioma invasion, further investigation will be necessary to better characterize EMT-like and its reverse MET-like processes occurring in GBM.

Targeting autophagy in GBM therapy is still a matter of debate; autophagy induction has been observed in GBM in response to radio- and temozolomide-based therapy and even though a number of clinical trials aimed at inhibiting autophagy execution, mainly by CQ, have been launched, others directed to inhibiting mTOR pathway, and thus activating autophagy, are ongoing.

Intriguingly, we observed that autophagy induction by nutrient starvation or by mTOR inhibition impairs migration and invasion of GBM cells, in line with other studies conducted on other cancer models. Further in-depth studies will be crucial to clearly dissect the autophagy role in GBM biology and to carefully evaluate autophagy modulation as therapeutic strategy to contrast GBM progression.

## Figures and Tables

**Table 1:** Autophagy and EMT.

Cell/Tissue	Autophagy Modulation	Effect on EMT on EMT	Mechanism	References
HCC cells	Induction by starvation Inhibition by ATG KD or CQ	Induction Inhibition	activation of TGFβ/Smad3-dependent and cAMP/PKA/CREB signalling	[108,127,128,129]
Colorectal cancer cells	Induction by mTOR inhibition or by ALS treatment Inhibition by BECN1 knockdown	Inhibition Inhibition	decreased activation of RhoA and Rac1 unknown	[122,130,131]
Ovarian cancer cells	Induction by Danu treatment	Inhibition	unknown	[123]
Non tumorigenic hepatocytes	Induction by starvation + TGF β_1_ Inhibition by BECN1 or ATG7 KD	Inhibition Induction	Snail degradation unknown	[126]
NPC cells	Induction by Cisplatin	Induction	unknown	[132]
NSCL cells	Induction by TGFβ_1_ treatment	Induction	unknown	[133]
Lung adenocarcinoma cells	Induction by MSCs co-colture	Induction	Snail up-regulation	[134]
Endometrial cells	Induction by Hypoxia	Induction	unknown	[135]
Uroepithelial cells	Induction by DBP exposure or starvation	Induction	E-cadherin degradation or TGFβ1/Smad3 pathway activation	[136,137]
Kidney podocytes	Inhibition by V-ATPase	Inhibition	Reduction of p62 phosphorylation	[138]
MEFs, keratinocytes, melanoma cells	Inhibition by ATG KD	Induction	p62-mediated Twist stabilization	[139]
Breast	Activation by DEDD overexpression	Inhibition	Snail and Twist degradation	[140]
Gastric cancer cells and tissue	Inhibition by BECN1 KD	Inhibition	ROS-NFκB-HIF-1α pathway activation	[141]

3-MA, 3-methyladenine; ALS, Alisertib (Aurora kinase A inhibitor); ATG, autophagy related gene; Baf, bafilomycin; BECN1, Beclin1; cAMP, cyclic adenosine monophosphate; CQ, chloroquine; CREB, cAMP responsive element binding; Danu, Danusertib; DBP, n-butyl phthalate; DEDD, death effector domain containing; DRAM1, DNA damage-regulated autophagy modulator 1; EMT, epithelial to mesenchymal transition; HCC, hepatocarcinoma cells; HIF-1α, hypoxia-inducible factor 1; KD, knockdown; MEFs, mouse embryonic fibroblasts; MSC, mesenchymal stem cell; NF- κB, nuclear factor kappa beta; NPC, nasopharyngeal carcinoma; NSCL, Non-small cell lung; PI3KC3, phosphatidylinositol 3-kinase; PKA, protein kinase A; Rac1, Ras-related C3 botulinum toxin substrate 1; RhoA, Ras homolog gene family, member A; ROS, reactive oxygen species; Smad3, small mother against decapentaplegic3; SQSTM1, sequestosome 1; TGFβ, Transforming growth factor beta; V-H-ATPase, vacuolar-type H^+^-adenosine triphosphatase.
